# Human Epidermal Growth Factor Receptor 2-Positive (HER2+) Early Breast Cancer Treatment and Outcomes by Risk of Recurrence: A Retrospective US Electronic Health Records Study

**DOI:** 10.3390/cancers17111848

**Published:** 2025-05-31

**Authors:** Reshma Mahtani, Simon M. Collin, Ziyu Tan, Chintal H. Shah, Basirat Adeyemi, Sophie Davies, Ellie John, Gregory Vidal

**Affiliations:** 1 Miami Cancer Institute, Baptist Health South Florida, Miami, FL 33176, USA; rmahtani@baptisthealth.net; 2 Oncology Outcomes Research (O2R), Evidence Generation, Publications, and Partnerships, Global Medical Affairs, Oncology Business Unit, AstraZeneca, Cambridge CB2 0AA, UK; ziyu.tan@astrazeneca.com; 3 Oncology Outcomes Research (O2R), Evidence Generation, Publications, and Partnerships, Global Medical Affairs, Oncology Business Unit, AstraZeneca, Gaithersburg, MD 20878, USA; chintal.shah@astrazeneca.com; 4 US Medical Affairs, Oncology Business Unit, AstraZeneca, Gaithersburg, MD 20878, USA; basirat.adeyemi@astrazeneca.com; 5 Global Medical Affairs, Oncology Business Unit, AstraZeneca, Cambridge CB2 0AA, UK; sophie.davies5@astrazeneca.com; 6 Global Medical Affairs Payer Biometrics, Statistics, Oncology Business Unit, AstraZeneca, Cambridge CB2 0AA, UK; ellie.john@astrazeneca.com; 7 West Cancer Center Research Institute and Lee S. Schwartzberg Research Center, Germantown, TN 38138, USA; gvidal@westclinic.com

**Keywords:** breast cancer, early stage, high-risk, HER2-positive, secondary data, observational study, treatment patterns, neoadjuvant therapy, adjuvant therapy, clinical outcomes

## Abstract

**Background/Objectives**: This study analyzed real-world data to assess patient characteristics, treatment patterns, and clinical outcomes in patients with human epidermal growth factor receptor 2-positive (HER2+) early breast cancer (eBC), as data for this patient group are limited. **Methods**: This was a retrospective observational cohort study of patients in a US electronic health record-derived deidentified database. Patient characteristics, treatment patterns, and clinical outcomes were analyzed overall and by risk of recurrence (high-risk versus non-high-risk). **Results**: Of 1290 patients, 366 (28.4%) were classified as high-risk and 924 (71.6%) as non-high-risk. At 5 years, high-risk versus non-high-risk eBC had a lower probability of invasive disease-free survival (72.3% [95% CI: 66.8–77.1%] vs. 80.7% [95% CI: 77.6–83.5%]), distant recurrence-free survival (78.7% [95% CI: 74.1–83.6%] vs. 89.3% [95% CI: 86.9–91.7%]), and overall survival (86.9% [95% CI: 82.3–90.4%] vs. 91.8% [95% CI: 89.4–93.7%]), and a shorter time to mBC diagnosis (22.6 vs. 34.1 months, respectively). Neoadjuvant therapy use increased from 18.2% in 2011–2013 to 67.3% in 2018–2021 in high-risk eBC and from 4.1% to 34.4% in non-high-risk eBC. However, 32.7% of eligible patients with high-risk eBC did not receive neoadjuvant therapy in 2018–2021. Use of post-neoadjuvant and adjuvant therapy increased in high-risk (but not non-high-risk) eBC from 81.8% in 2011–2013 to 91.8% in 2018–2021. **Conclusions**: High-risk HER2+ eBC manifested more aggressively than non-high-risk eBC. Utilization of neoadjuvant and (post-neo)adjuvant therapy increased over time. However, despite guideline recommendations, uptake of neoadjuvant therapy remains suboptimal.

## Simple Summary

At present, there is limited information on real-world treatment patterns and how outcomes might differ by risk of recurrence in patients with human epidermal growth factor receptor 2-positive (HER2+) early breast cancer (eBC). This study aimed to describe the use of neoadjuvant and (post-neo)adjuvant therapies and clinical outcomes by risk of recurrence in a real-world population of patients with HER2+ eBC from the US. This study confirmed that high- versus non-high recurrence risk in HER2+ eBC is associated with a shorter time from eBC diagnosis to metastatic breast cancer diagnosis, invasive disease, and distant recurrence, and a lower probability of overall survival. Although the use of neoadjuvant and (post-neo)adjuvant therapy has increased over time in patients with high-risk HER2+ eBC, there remains a need for improvement in treatment outcomes and for more effective therapies for all patients with HER2+ eBC.

## 1. Introduction

In 2020, breast cancer (BC) was the most commonly diagnosed cancer in women worldwide, and the second most common cancer overall. There were more than 2.31 million new cases in 2022 [1]. Around 90% of women diagnosed with BC in the US are diagnosed with early BC (eBC) [2]. It is estimated that human epidermal growth factor receptor 2-positive (HER2+) BC, which is defined as amplification and/or protein overexpression of the HER2 gene, accounts for nearly 20% of all BC cases [3,4,5]. If untreated with appropriate HER2-directed therapy, or in cases where eBC presents with extensive local disease burden and/or lymph node involvement, HER2+ BC is associated with a poor prognosis, including high recurrence rates, an increased likelihood of brain metastases, and high mortality [6,7].

The currently recommended standard of care (SOC) for HER2+ eBC in the neoadjuvant setting consists of a multi-agent regimen of dual-HER2-directed therapy (trastuzumab + pertuzumab) with a chemotherapy backbone (docetaxel or paclitaxel + carboplatin is the recommended SOC in the US, although anthracycline and cyclophosphamide with a taxane may also be considered) [8,9,10]. In the post-neoadjuvant setting, patients receive individualized treatment according to their pathologic complete response (pCR) status and tumor burden [11,12]. Despite the improvements that HER2-directed therapies have made to eBC survival and recurrence rates, patients with HER2+ eBC remain at risk of recurrence, even when treated with SOC therapies [7,13,14]. It has been reported that up to 25% of patients with HER2+ eBC will suffer disease recurrence within 10 years, even when treated with HER2-directed therapies (dependent on initial stage, tumor biology, and initial treatment received) [7].

In an effort to improve treatment outcomes, risk factors for recurrence of disease in patients with HER2+ eBC are being identified [7] and treatment guidelines have evolved to recommend the use of neoadjuvant therapy in the patients with HER2+ eBC considered to be at high-risk of recurrence [10]. At present, there is limited information available regarding real-world treatment patterns and how treatment outcomes might be affected by the risk of recurrence in patients with HER2+ eBC. Therefore, we performed an analysis of real-world data from patients in the US with HER2+ eBC to assess patient characteristics, treatment patterns, and clinical outcomes overall and by risk of recurrence. We defined patients as having either high-risk or non-high-risk eBC based on pathologic and/or clinical staging. We aim to address a key data gap in the current treatment landscape and provide supporting data for the uptake of new treatment options for patients diagnosed with HER2+ eBC.

## 2. Materials and Methods

### 2.1. Data Source

This study used the US nationwide Flatiron Health electronic health record-derived deidentified database, consisting of processed longitudinal patient-level medical record data (structured and unstructured). Data are curated via technology-enabled abstraction and provide information on patient demographics, diagnosis (e.g., staging, histopathology, and biomarkers), treatment, and outcomes (e.g., mortality) [15,16]. During the study period, the deidentified data originated from approximately 280 cancer clinics, representing ~800 sites of care, primarily community (~80%) and academic (~20%) oncology settings, broadly distributed across the US. Limited National Comprehensive Cancer Network member institutions also participate in Flatiron Health data collection [15,16]. The data are deidentified and subject to obligations to prevent reidentification and protect patient confidentiality. Since the study only used deidentified patient records and did not involve the collection, use, or transmittal of individually identifiable data, Institutional Review Board approval to conduct this study was not required.

### 2.2. Study Design

**Figure 1:**
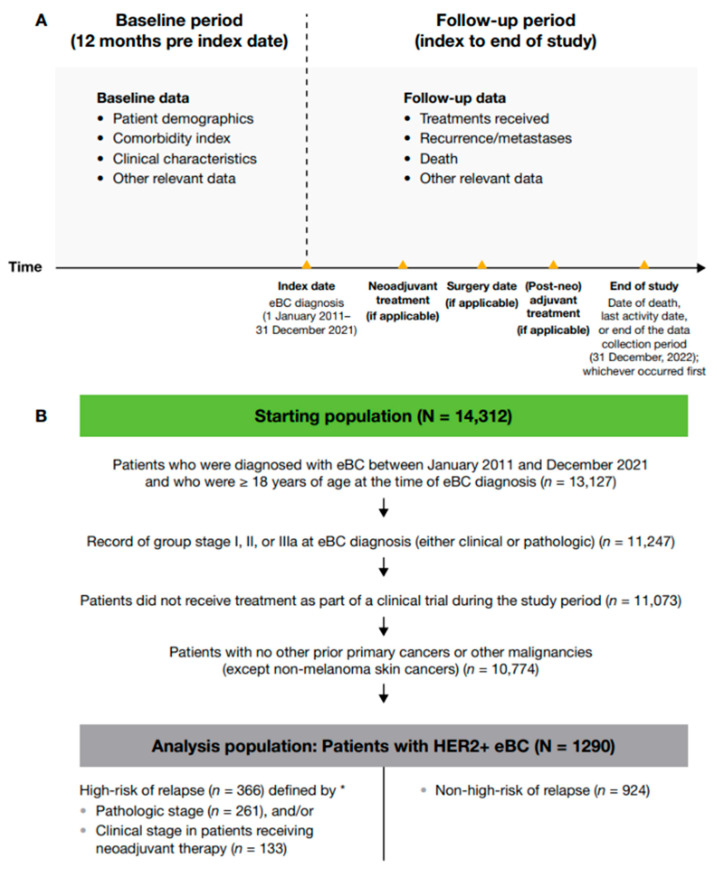
(**A**) Study design and (**B**) patient attrition. * High-risk eBC was defined using pathologic staging as follows: patients with nodal involvement (T0–4, N1–3, M0); or tumor size > 5 cm (T3–4, N0, M0); or nodal involvement; or by clinical stage IIb, IIc, IIIa, IIIb, or IIIc in patients who received neoadjuvant therapy; 28 patients met the criteria for high-risk eBC by both pathologic staging and clinical stage. eBC, early breast cancer; HER2+, human epidermal growth factor receptor 2-positive.

A retrospective observational cohort study of patients diagnosed with HER2+ eBC (i.e., stage I, II, or III) between 1 January 2011, and 31 December 2021, was conducted using the Flatiron Health deidentified database. Patient data were categorized by the date of eBC diagnosis: 2011–2013, 2014–2017, or 2018–2021. Patients were identified through the electronic data from their HER2+ eBC diagnosis until their date of death, if available, or last activity date if there was no record of the patient’s death (Figure 1).

Baseline data, including patient demographics, comorbidities, and clinical characteristics, were collected for the 12 months before index date (i.e., date of eBC diagnosis). Follow-up data were gathered from index to date of death, last activity date, or end of the data collection period (31 December 2022), whichever occurred first. Treatment data included receipt of therapy after surgery (post-neoadjuvant therapy if the patient received neoadjuvant therapy before surgery or adjuvant therapy if the patient received surgery only; these two modalities are hereafter referred to jointly as (post-neo)adjuvant therapy). Clinical outcomes included invasive disease-free survival (IDFS), overall survival (OS), and distant recurrence-free survival (DRFS). IDFS was defined as the time from date of initial diagnosis (if no surgery occurred) or the date of definitive BC surgery until the earliest date of invasive locoregional recurrence, invasive distant recurrence (including contralateral BC), second primary malignancy, or death. If no IDFS event of interest was observed, patients were censored at the date of last contact or study end date, whichever occurred first. DRFS was defined as the time from date of initial diagnosis (if no surgery occurred) or the date of definitive BC surgery until the earliest date of distant recurrence (including contralateral BC) or death. If no DRFS event of interest was observed, patients were censored at the date of last contact or study end date, whichever occurred first.

### 2.3. Study Sample

Adult patients (≥ 18 years at diagnosis) who were diagnosed with HER2+ eBC between 1 January 2011, and 31 December 2021, were eligible for inclusion. Patients participating in clinical trials were excluded. A primary cancer or malignancy other than eBC (except non-melanoma skin cancers) was exclusionary unless curatively treated with no evidence of disease for ≥ 3 years prior to eBC diagnosis.

### 2.4. Tumor Staging

Patients were grouped according to their risk of disease recurrence (high versus non-high). High-risk eBC was defined using two overlapping criteria: in all patients, pathologic stage showing nodal involvement (T0–4, N1–3, M0), or tumor size > 5 cm (T3–4, N0, M0); in patients who received neoadjuvant therapy, clinical group stage IIb, IIc, IIIa, IIIb, or IIIc, irrespective of their (post-neo)adjuvant stage. A sensitivity analysis was conducted, expanding the high-risk criteria for patients who had received neoadjuvant therapy to include patients with stage IIA disease. Any patients who did not meet these criteria were classified as having non-high-risk eBC. Of note, pCR data were missing for 25% of the clinical stage IIb+ group.

### 2.5. Objectives

The primary objectives were to describe the demographic and clinical characteristics and treatment patterns of patients with HER2+ eBC, overall and by their risk of disease recurrence. The secondary objective was to assess the clinical outcomes of patients with HER2+ eBC, overall and by risk status. The exploratory objective was to characterize trends in treatment patterns over time in patients with HER2+ eBC, overall and by risk status.

### 2.6. Statistical Methods

All analyses were descriptive, with no formal comparisons between groups, and were conducted using SAS (version 9.4) or R. Clinical, while demographic characteristics were summarized descriptively. Treatment patterns were summarized using frequency counts and percentages. Kaplan–Meier (KM) methods were used to summarize survival endpoints, which included a graph depicting the survival curve and estimates of median survival, together with their 95% confidence intervals (CIs). Estimates of survival probabilities at 6-monthly landmark intervals (6, 12, 18 months, etc.) and corresponding 95% CIs were reported for each time-to-event outcome (IDFS, OS, and DRFS). KM estimates of duration of treatment and the landmark survival rate at 5 years were reported for each time-to-event outcome, along with the 95% CIs. The 5-year interval was chosen based on the average follow-up time of censored patients.

Pathologic complete response (pCR) was coded in the ‘pathologic group staging’ variable within the database, which combines the results of the clinical staging (physical exam, imaging tests) with surgical results. pCR is captured as explicitly stated by the physician or in a pathology report. For example, if a pathology report indicates that there is no residual invasive disease in the breast or axillary lymph nodes after treatment, the abstractor will record ‘Patient had a pCR’ as the pathologic group staging value for this patient.

## 3. Results

### 3.1. Patient Population

Of 13,127 adult patients in the Flatiron Health real-world database who were diagnosed with eBC during the study period, 1290 met the eligibility criteria for this study (Figure 1); of these, 351 were from the period 2011–2013, 515 from 2014–2017, and 424 from 2018–2021. In total, 366 (28.4%) patients were classified as having a high-risk of disease recurrence (261 by pathologic stage criteria and 133 by clinical stage criteria in those who received neoadjuvant therapy; 28 patients met criteria for both clinical and pathologic high-risk) and 924 (71.6%) as non-high-risk.

### 3.2. Patient Demographics and Baseline Disease Characteristics

Patients diagnosed with HER2+ eBC had a mean age at diagnosis of 57.6 years and were predominantly White (63.3%) (Table 1; see Table A1 in Appendix A for demographic characteristics in the sensitivity analysis). The majority of patients in the high-risk HER2+ group were postmenopausal (57.9%), as were the majority of the patients in the non-high-risk group (66.8%). 

The median time from HER2+ eBC diagnosis to HER2+ metastatic BC (mBC) diagnosis was 22.6 (IQR: 17.8–36.1) months in the high-risk group and 34.1 (IQR: 20.0–56.6) months in the non-high-risk group. The majority of patients had invasive histology in both the high-risk HER2+ group (94.3% invasive ductal carcinoma [IDC] and 3.3% invasive lobular carcinoma [ILC]) and the non-high-risk group (92.3% IDC and 3.7% ILC).

### 3.3. Treatment Patterns in Patients with HER2+ eBC

Almost all (97.3%) patients with HER2+ eBC had primary surgery, most commonly a unilateral lumpectomy (52.1%) or a unilateral mastectomy (24.4%). Of the patients with high-risk eBC, 34.7% had a unilateral lumpectomy and 35.0% had a unilateral mastectomy versus 53.9% and 18.0% of patients with non-high-risk eBC, respectively; among patients for whom germline BRCA test data were available (34.3%, 443/1290), 4.0% (5/126) of patients with high-risk eBC and 4.4% (14/317) of patients with non-high-risk eBC were non-wild type. Over the whole study period (2011–2021), compared with patients with high-risk eBC, patients with non-high-risk eBC were more likely to have surgery only (17.2% vs. 11.2%) or surgery plus adjuvant therapy (59.2% vs. 44%); patients with high-risk eBC were more likely to receive neoadjuvant therapy followed by surgery and (post-neo)adjuvant therapy than patients with non-high-risk eBC (43.7% vs. 19.8%) (Table 2; see Table A2 in the Appendix A for treatment breakdown in sensitivity analysis).

**Figure 2:**
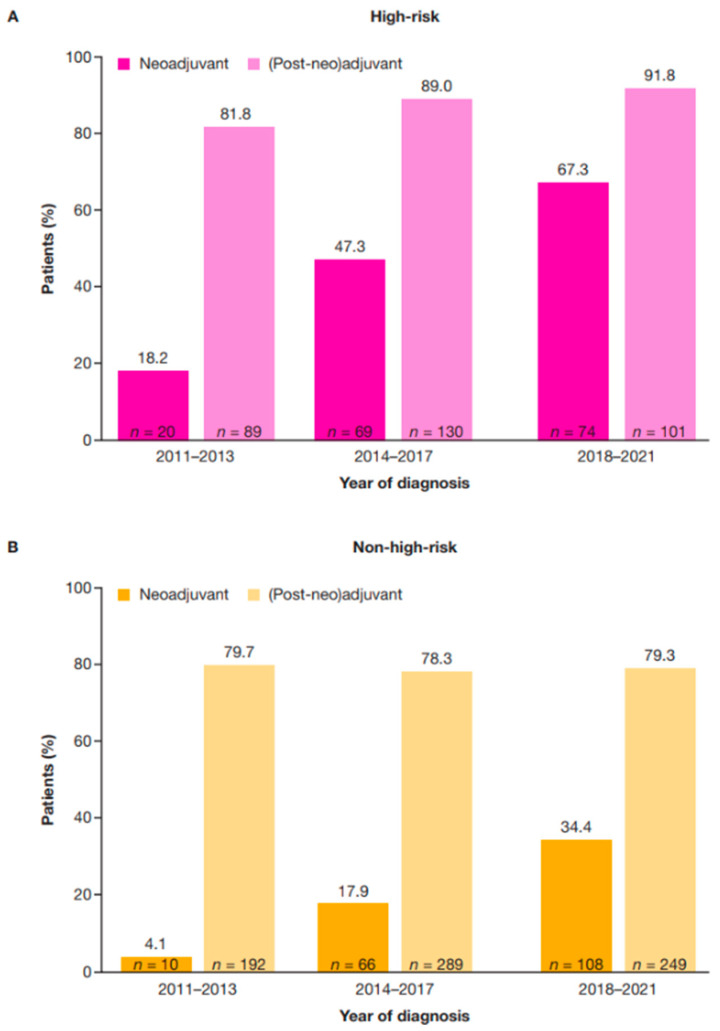
Neoadjuvant and (post-neo)adjuvant treatment by diagnosis year in patients with high-risk (**A**) and non-high-risk (**B**) HER2+ eBC. High-risk eBC was defined using pathologic staging as follows: patients with nodal involvement (T0–4, N1–3, M0); or tumor size > 5 cm (T3–4, N0, M0); or nodal involvement; or by clinical stage IIb, IIc, IIIa, IIIb, or IIIc in patients who received neoadjuvant therapy. eBC, early breast cancer; HER2+, human epidermal growth factor receptor 2-positive.

Use of neoadjuvant therapy increased over time in both high-risk and non-high-risk populations. For patients diagnosed between 2018 and 2021, neoadjuvant treatment was received by around two-thirds (67.3%) of those classified as having high-risk eBC, and by one-third (34.4%) of those classified as having non-high-risk eBC. This was an increase from 18.2% of high-risk and 4.1% of non-high-risk eBC diagnosed in the period 2011–2013 and 47.3% of high-risk and 17.9% of non-high-risk eBC diagnosed in the period 2014–2017 (Figure 2).

Among patients at high-risk who received neoadjuvant therapy, 43.5% (70/161) reached pCR; the corresponding proportion among patients with non-high-risk HER2+ eBC was 58.2% (106/182). Among patients with high-risk eBC who received neoadjuvant therapy, 63.2% (103/163) were estrogen receptor-positive (ER+), 47.2% (77/163) were progesterone receptor-positive (PR+), and 66.9% (109/163) were hormone receptor-positive (HR+) compared with 74.4% (151/203) ER+, 57.6% (117/203) PR+, and 76.8% (156/203) HR+ among patients with high-risk eBC who did not receive neoadjuvant therapy (Table 3).

Per the methodology described above, the term (post-neo)adjuvant therapy is used herein if the patient received therapy before surgery; adjuvant therapy is used if the patient received surgery only. Use of (post-neo)adjuvant therapy increased from 81.8% of patients with high-risk HER2+ eBC in the period 2011–2013, to 91.8% of patients in the period 2018–2021; use in non-high-risk eBC was consistent across the period from 2011–2013 to 2018–2021 (Figure 2). A higher proportion of patients who received (post-neo)adjuvant therapy were HR+ in the high-risk (75.1%; 241/321) and non-high-risk (78.0%; 569/730) groups compared with those who did not receive (post-neo)adjuvant therapy in the high-risk (53.3%; 24/45) and non-high-risk groups (54.6%; 106/194) (Table 4).

When stratified by HR status, neoadjuvant therapy was received by a higher proportion of patients with high-risk HER2+ eBC who were hormone receptor-negative (HR−) than those who were hormone receptor-positive (HR+), whereas the opposite pattern was seen with (post-neo)adjuvant therapy, across all diagnosis periods (2011–2013, 2014–2017, and 2018–2021) (Table 5).

Across all subgroups (risk status, diagnosis period, HR status), receipt of a HER2-directed neoadjuvant therapy increased over time (increasing from 14.7% of high-risk and 3.3% of non-high-risk HER2+ eBC in 2011–2013 to 64.5% and 33.8%, respectively, by 2018–2021). (Post-neo)adjuvant therapy was received by most patients with high-risk HER2+ eBC (> 87%) across all subgroups, with the exception of patients who were HR− and diagnosed between 2011 and 2013, where 55.2% received (post-neo)adjuvant therapy (Table 5; see Table A3 and Table A4 in Appendix A for full breakdown of treatments). Approximately two-thirds of patients who were HR+ received endocrine therapy in the (post-neo)adjuvant setting in all diagnosis periods. The proportion of patients who received HER2-directed therapy in the (post-neo)adjuvant setting increased over time from 55.0% (high-risk) and 45.6% (non-high-risk) for patients diagnosed between 2011 and 2013 to 86.4% (high-risk) and 68.8% (non-high-risk) for those diagnosed between 2018 and 2021. Conversely, use of (post-neo)adjuvant chemotherapy and endocrine therapy was consistent over time regardless of risk status.

Median time (IQR) from diagnosis to neoadjuvant therapy for patients with high-risk HER2+ eBC who received neoadjuvant therapy was 29 days (23–43 days), compared with 34 days (27–44 days) in patients with non-high-risk HER2+ eBC. Median duration (IQR) of neoadjuvant therapy was 145 days (132–163 days) in high-risk HER2+ eBC, compared with 141 days (126–155 days) in non-high-risk HER2+ eBC. Median time (IQR) between diagnosis and surgery was 76 days (26–180 days) for patients with high-risk and 36 days (15–151 days) for patients with non-high-risk HER2+ eBC (overall data not stratified by receipt of neoadjuvant therapy). Median time (IQR) between surgery and (post-neo)adjuvant therapy was 31 days (17–71 days) for patients with high-risk and 44 days (24–84 days) for patients with non-high-risk HER2+ eBC (Table 6).

### 3.4. Clinical Outcomes

**Figure 3:**
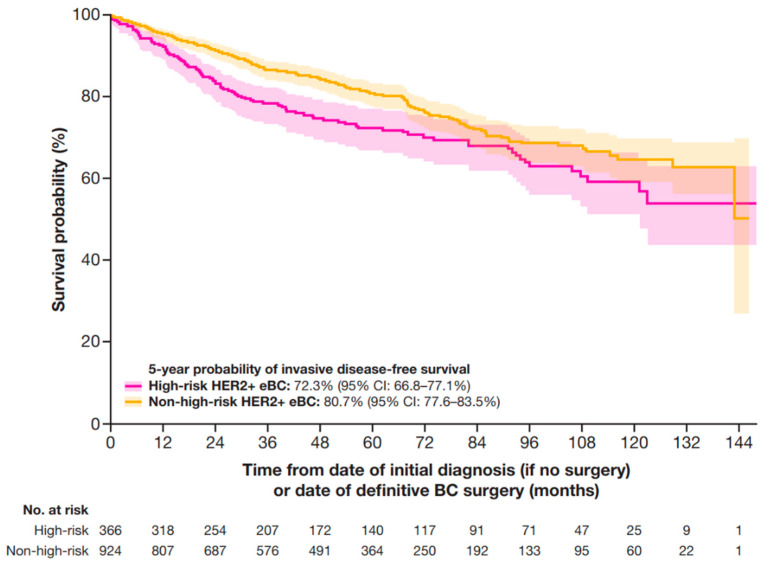
IDFS in patients with HER2+ eBC. High-risk eBC was defined using pathologic staging as follows: patients with nodal involvement (T0–4, N1–3, M0); or tumor size > 5 cm (T3–4, N0, M0); or nodal involvement; or by clinical stage IIb, IIc, IIIa, IIIb, or IIIc in patients who received neoadjuvant therapy; IDFS was defined as time from date of initial diagnosis (if no surgery) or date of definitive BC surgery until the earliest date of invasive locoregional recurrence, invasive distant recurrence, second primary malignancy, or death. If no event of interest was observed, patients were censored at the date of last contact or study end date, whichever occurred first. BC, breast cancer; CI, confidence interval; eBC, early BC; HER2+, human epidermal growth factor receptor 2-positive; IDFS, invasive disease-free survival.

The median (IQR) follow-up time for this study was 5 years (2.7–7.3 years). At 5 years, the IDFS probability was 72.3% (95% CI: 66.8–77.1%) for patients at high-risk and 80.7% (95% CI: 77.6–83.5%) for patients at non-high-risk (Figure 3).

**Figure 4:**
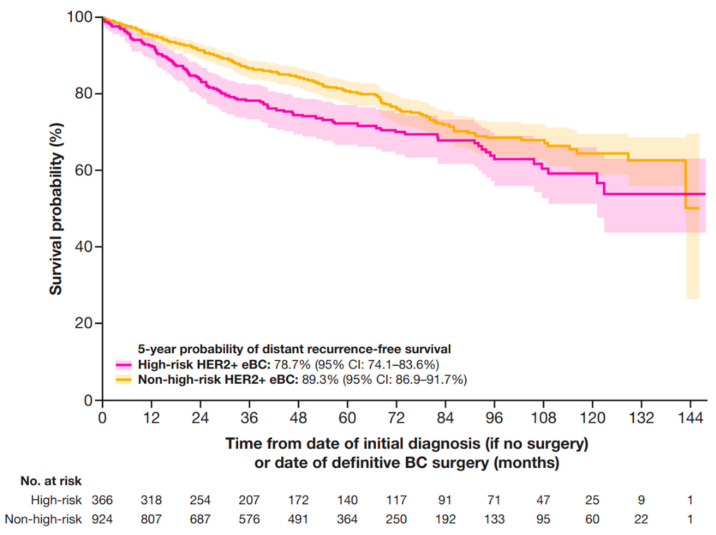
DRFS in patients with HER2+ eBC. DRFS was defined as the time from date of initial diagnosis (if no surgery occurred) or the date of definitive BC surgery until the earliest date of distant recurrence (including contralateral BC) or death. High-risk eBC was defined using pathologic staging as follows: patients with nodal involvement (T0–4, N1–3, M0); or tumor size > 5 cm (T3–4, N0, M0); or nodal involvement; or by clinical stage IIb, IIc, IIIa, IIIb, or IIIc in patients who received neoadjuvant therapy. BC, breast cancer; CI, confidence interval; DRFS, distant recurrence-free survival; eBC, early BC; HER2+, human epidermal growth factor receptor 2-positive.

At 5 years, the DRFS probability was 78.7% (95% CI: 74.1–83.6%) for patients with high-risk HER2+ eBC and 89.3% (95% CI: 86.9–91.7%) in patients with non-high-risk HER2+ eBC (Figure 4).

**Figure 5:**
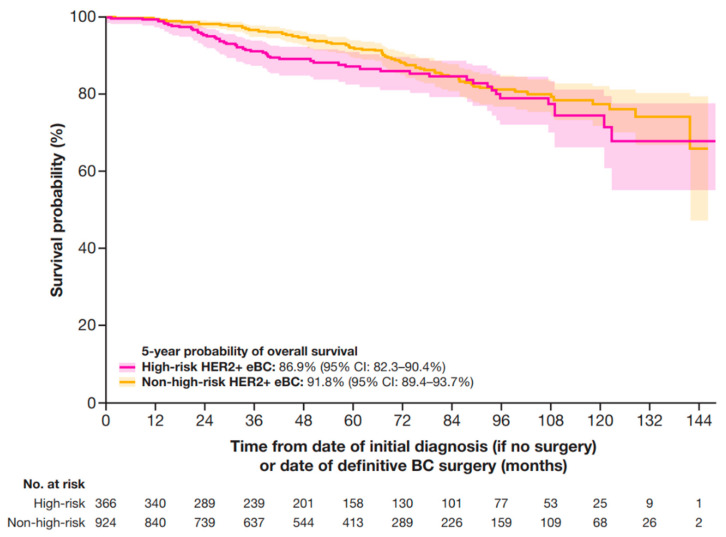
OS in patients with HER2+ eBC. High-risk eBC was defined using pathologic staging as follows: patients with nodal involvement (T0–4, N1–3, M0); or tumor size > 5 cm (T3–4, N0, M0); or nodal involvement; or by clinical stage IIb, IIc, IIIa, IIIb, or IIIc in patients who received neoadjuvant therapy. CI, confidence interval; eBC, early breast cancer; HER2+, human epidermal growth factor receptor 2-positive; OS, overall survival.

At 5 years, the OS probability was 86.9% (95% CI: 82.3–90.4%) for patients at high-risk and 91.8% (95% CI: 89.4–93.7%) for patients at non-high-risk (Figure 5).

**Figure A1:**
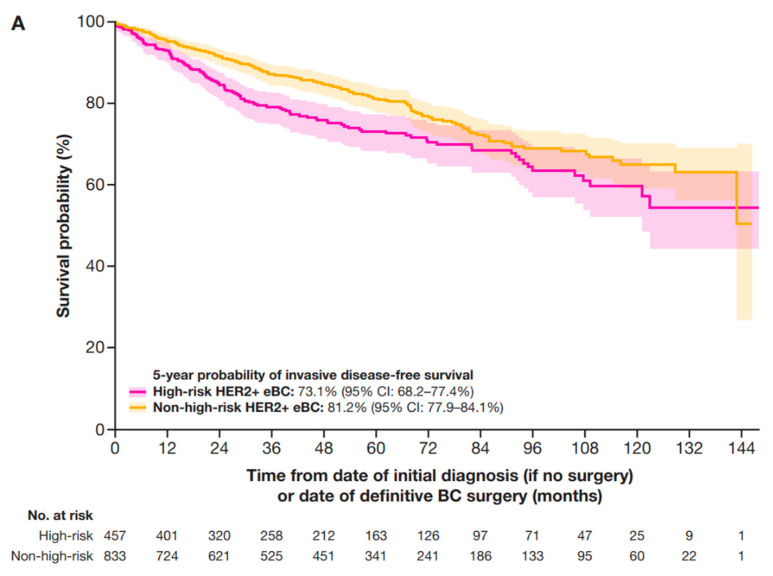

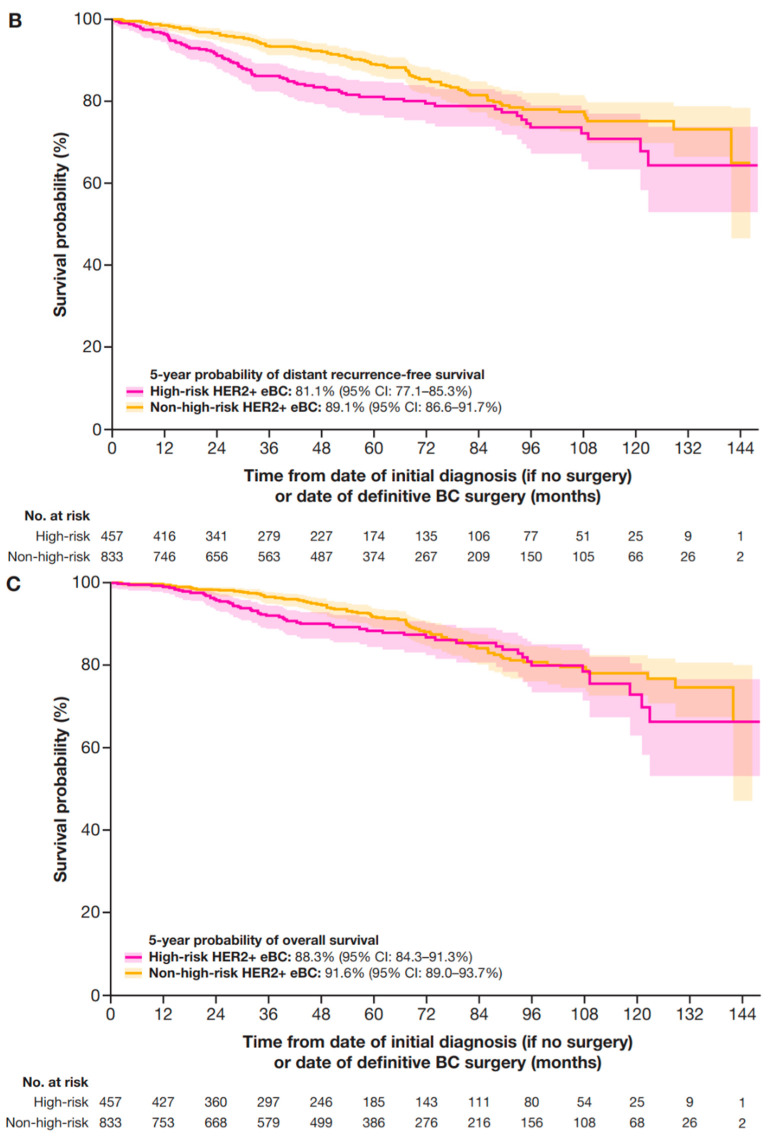
Sensitivity analyses of 5-year outcomes with stage IIa included in the high-risk criteria for (**A**) IDFS, (**B**) DRFS, and (**C**) OS. CI, confidence interval; DRFS, distant recurrence-free survival; eBC, early breast cancer; HER2+, human epidermal growth factor receptor 2-positive; IDFS, invasive disease-free survival; OS, overall survival.

In a sensitivity analysis that included patients with stage IIA HER2+ eBC (who had received neoadjuvant therapy) in the high-risk group (Table A3), 5-year IDFS, DRFS, and OS probabilities were 73.1% (95% CI: 68.2–77.4%), 81.1% (95% CI: 77.1–85.3%), and 88.3% (95% CI: 84.3–91.3%), respectively (Figure A1).

## 4. Discussion

This study aimed to address a data gap in the HER2+ eBC treatment landscape by providing data on patient demographics, real-world treatment patterns, and outcomes in patients diagnosed with HER2+ eBC. In addition to overall treatment patterns in HER2+ eBC, this study aimed to explore how treatment patterns may differ by risk of disease recurrence, identified at diagnosis and defined by clinical and/or pathologic stage(s) at diagnosis.

Patients with high-risk HER2+ eBC (defined in this study using pathologic staging as follows: patients with nodal involvement [T0–4, N1–3, M0]; or tumor size > 5 cm [T3–4, N0, M0]; or nodal involvement; or by clinical stage IIb, IIc, IIIa, IIIb, or IIIc in patients who received neoadjuvant therapy) were of particular interest in this study owing to evidence that, even when treated with HER2-directed therapies, up to 20% of patients with HER2+ eBC will experience disease recurrence within 8 years [17]. A recurrence of HER2+ BC, which has a more aggressive biology and is prone to metastasis, is considered incurable in the setting of distant recurrence [18]. This highlights the critical importance of identifying patients at high-risk of recurrence and initiating appropriate treatment, with the intention of preventing recurrence. In this study, 28% of the patients diagnosed with HER2+ eBC were classified as high-risk and 72% as non-high-risk. As expected, patients with high-risk HER2+ eBC experienced poorer outcomes when compared with patients with non-high-risk HER2+ eBC, as indicated by the clinical outcomes analyzed. Specifically, the median time from eBC to mBC diagnosis for patients with high-risk HER2+ eBC was 23 months compared with 34 months in patients with non-high-risk HER2+ eBC. Patients with high-risk HER2+ eBC also experienced numerically lower 5-year IDFS, DRFS, and OS probabilities of 72%, 79%, and 87%, respectively, compared with patients at non-high-risk, who had 5-year probabilities of 81%, 89%, and 92%, respectively.

A lower proportion of patients with high-risk HER2+ eBC who received neoadjuvant therapy reached pCR, compared with patients with non-high-risk HER2+ eBC who received neoadjuvant therapy (44% vs. 58%). Residual cancer burden and non-pCR are associated with an increased risk of disease recurrence [7], and achievement of pCR after neoadjuvant therapy may be prognostic as it appears to be a reasonable surrogate marker of long-term outcomes in patients with HER2+ BC [19]. Patients with residual disease are candidates for (post-neo)adjuvant/adjuvant HER2-directed therapy, because this has demonstrated an OS benefit [17].

A further aim of this study was to describe the treatment landscape by exploring data on treatment patterns from 2011 to 2021. This study provides evidence of an upward trend in the use of neoadjuvant therapy in both high-risk and non-high-risk HER2+ eBC over this period. Approximately two-thirds (67%) of patients with high-risk HER2+ eBC and one-third (34%) of patients with non-high-risk HER2+ eBC received neoadjuvant therapy in the period 2018–2021. This was an increase from 18% of patients with high-risk HER2+ eBC and 4% of patients with non-high-risk HER2+ eBC diagnosed between 2011 and 2013, which aligns with the evolution of treatment guidelines that currently recommend the use of neoadjuvant therapy in patients with HER2+ eBC considered to be at high-risk of recurrence [10]. Nevertheless, one-third (33%) of patients with high-risk HER2+ eBC did not receive neoadjuvant therapy in the period 2018–2021, highlighting a clear area of unmet need in the treatment of HER2+ eBC.

An increase in the use of neoadjuvant treatment also aligns with the advent of pertuzumab, which was approved in 2013 for use in combination with trastuzumab and docetaxel as a neoadjuvant treatment in patients with HER2+ eBC [20]. The publication of pivotal studies regarding the efficacy and safety of pertuzumab in the neoadjuvant treatment of HER2+ eBC also likely contributed to this upward trend from 2011 to 2021. For instance, in 2012, the NeoSphere study demonstrated that neoadjuvant pertuzumab with trastuzumab and docetaxel resulted in significantly improved pCR rates compared with those given trastuzumab plus docetaxel [21].

This study also provides evidence of a slight upward trend in (post-neo)adjuvant therapy in patients with high-risk HER2+ eBC between the periods 2011–2013 and 2018–2021 (82% vs. 92%). On the other hand, use in patients with non-high-risk HER2+ eBC was consistent between 2011 and 2021. The upward trend in (post-neo)adjuvant therapy in patients with high-risk HER2+ eBC may be explained by the approval of trastuzumab emtansine and the publication of key data from the KATHERINE study in 2019 [22]. This study found that, in patients with HER2+ eBC who had residual invasive disease after completion of neoadjuvant therapy, the risk of recurrence was 50% lower when patients received adjuvant treatment with trastuzumab emtansine rather than the SOC at the time, trastuzumab monotherapy [22]. Similarly, data from ExteNET published in 2017 demonstrated that 1 year of neratinib with chemotherapy and trastuzumab in the (post-neo)adjuvant setting reduced the number of clinically relevant BC recurrences in patients with HER2+ eBC [23]. Interestingly, recent > 8-year follow-up data from ExteNET and APHINITY (neratinib and pertuzumab with trastuzumab, respectively) found no improvements in OS in patients treated with HER2-directed agents in the adjuvant setting [24,25]. In contrast, recent results from the final KATHERINE IDFS and OS analyses may result in future increases in (post-neo)adjuvant therapy as 7-year IDFS rates improved by 14% and OS rates improved by 5% when patients received adjuvant trastuzumab emtansine instead of the SOC at the time, trastuzumab [17]. This makes it difficult to predict how the treatment landscape may change following the patterns described in this study. Future research may focus on long-term OS in order to explore the efficacy of treatment in the (post-neo)adjuvant setting. Recent publications may continue to shape treatment patterns in the neoadjuvant and (post-neo)adjuvant settings. For example, the final analysis of the BERENICE study demonstrated high rates of 5-year event-free survival and OS in patients with HER2+ eBC who received neoadjuvant and adjuvant pertuzumab-trastuzumab-based therapy [26]. The introduction of new therapies and regimens, including novel HER2-directed modalities, may also shape the neoadjuvant and (post-neo)adjuvant treatment landscape in the near future.

The main strength of this study is the size of the real-world database and the length of outcome follow up (median 5 years), with the use of longitudinal data from 2011 to 2021, which allowed for reasonably precise estimations of outcomes and exploration of trends in the US treatment landscape for HER2+ eBC. Our results may be generalizable to community practices across the US, but other clinical settings may differ in terms of care provision and patient outcomes. It should be noted that certain treatments and procedures may have been underreported or not captured, particularly if provided outside the Flatiron Health network.

The main limitation of this study is that a number of factors can increase risk of cancer recurrence; therefore, any definition of a ‘high-risk’ patient group will be an over-simplification that imperfectly predicts individual outcomes [7]. Our definition was a composite of the previous clinical trial criteria of pathologic stage indicating nodal involvement or tumor size > 5 cm plus clinical stage IIb or higher in patients who received neoadjuvant therapy [27,28]. The T3 tumor size criterion is, however, stricter than the current T2 threshold for offering neoadjuvant therapy; nodal involvement and greater tumor burden are associated with higher rates of recurrence in eBC [29]. Similarly, tumors that are T3 or T4 are associated with up to 5% lower rates of pCR [30]. The clinical stage criterion, representing one-third of the high-risk group, captured patients whose cancer was more advanced at initial diagnosis regardless of subsequent response to neoadjuvant therapy. We might have included only those who did not experience pCR, but pCR data were missing for 25% of the clinical stage IIb+ group, which limited the potential for further analyses. Conversely, we explored less stringent high-risk criteria by expanding this group to include patients with clinical stage IIa. In this sensitivity analysis, IDFS, DRFS and OS for patients with high-risk eBC increased only slightly. Similarly, other risk factors could have been used to define a high-risk patient group [31], such as relatively low baseline levels of Ki-67 and tumor-infiltrating lymphocytes, a young age at diagnosis, and a higher body mass index [7], but this approach would yield more complicated and therefore potentially less replicable criteria restricted by data availability. A more in-depth analysis using causal models and inference based on a more complete dataset would represent a useful future project.

## 5. Conclusions

High-risk versus non-high-risk HER2+ eBC manifested aggressively in these patients, as indicated by the shorter time from eBC to mBC diagnosis, and the shorter IDFS, DRFS, and OS. In patients with high-risk and non-high-risk HER2+ eBC, the use of neoadjuvant and (post-neo)adjuvant therapy has increased over time. Despite this increase, and recent guideline recommendations, approximately one-third of patients with high-risk HER2+ eBC did not receive neoadjuvant therapy in 2018–2021. Although patients with high-risk HER2+ eBC experienced a poorer prognosis than those categorized as non-high-risk, there remains substantial margin for improvement in outcomes for all patients with HER2+ eBC, through early identification and prompt intervention with more effective therapies.

## Figures and Tables

**Table 1:** Demographic characteristics in patients with HER2+ eBC patients overall and stratified by high-risk status.

	Overall (N = 1290)	High-Risk * (*n* = 366)	Non-High-Risk (*n* = 924)
Age at diagnosis			
Mean (SD)	57.6 (12.8)	55.5 (13.5)	58.5 (12.4)
Median [min, max]	58.0 [23.0, 85.0]	56.0 [23.0, 84.0]	59.0 [23.0, 85.0]
Sex, *n* (%) ^†^			
Female	1281 (99.3)	≥ 360 (≥98)	918 (99.4)
Male	9 (0.7)	≤ 5 (≤ 1)	6 (0.6)
Race, *n* (%)			
White	817 (63.3)	223 (60.9)	594 (64.3)
Black	133 (10.3)	38 (10.4)	95 (10.3)
Asian	51 (3.9)	13 (3.6)	38 (4.1)
Other	178 (13.8)	59 (16.1)	119 (12.9)
Missing	111 (8.6)	33 (9.0)	78 (8.4)
Ethnicity, *n* (%)			
Hispanic/Latino	104 (8.1)	33 (9.0)	71 (7.7)
Non-Hispanic/Non-Latino	982 (76.1)	281 (76.8)	701 (75.9)
Missing	204 (15.8)	52 (14.2)	152 (16.5)
BMI at diagnosis, *n* (%) ^‡^			
Normal (18.5–< 25)	49 (3.8)	13 (3.6)	36 (3.9)
Overweight (25–< 30)	55 (4.3)	17 (4.6)	38 (4.1)
Obese (≥ 30)	66 (5.1)	23 (6.3)	43 (4.7)
Missing	1119 (86.7)	313 (85.5)	806 (87.2)
Underweight (< 18.5)	1 (0.1)	0 (0)	1 (0.1)
Region, *n* (%)			
South	500 (38.8)	138 (37.7)	362 (39.2)
Northeast	170 (13.2)	48 (13.1)	122 (13.2)
West	171 (13.3)	49 (13.4)	122 (13.2)
Midwest	165 (12.8)	43 (11.7)	122 (13.2)
Missing	284 (22.0)	88 (24.0)	196 (21.2)
Menopausal status, *n* (%)			
Premenopausal	340 (26.4)	120 (32.8)	220 (23.8)
Postmenopausal	829 (64.3)	212 (57.9)	617 (66.8)
Perimenopausal	33 (2.6)	10 (2.7)	23 (2.5)
Missing/not applicable/male	88 (6.8)	24 (6.6)	64 (6.9)
Months of follow up			
Mean (SD)	61.5 (35.8)	63.0 (36.5)	61.0 (35.5)
Median (IQR)	58.0 (31.9, 87.8)	58.2 (31.1, 94.7)	57.8 (32.4, 85.1)

* High-risk eBC was defined using pathologic staging as follows: patients with nodal involvement (T0–4, N1–3, M0); or tumor size > 5 cm (T3–4, N0, M0); or nodal involvement; or by clinical stage IIb, IIc, IIIa, IIIb, or IIIc in patients who received neoadjuvant therapy; ^†^ To prevent patient identification, values ≤ 5 are masked; ^‡^ BMI calculated as kg/m^2^. BMI, body mass index; IQR, interquartile range; SD, standard deviation.

**Table 2:** Treatment pathways from eBC diagnosis over the whole study period (2011–2021) stratified by risk status.

	High-Risk * *n* = 366	Non-High-Risk *n* = 924
Treatment pathway after diagnosis, *n* (%)		
No treatment	1 (0.3)	34 (3.7)
Neoadjuvant treatment only	0	0
Neoadjuvant treatment followed by surgery only	3 (0.8)	1 (0.1)
Surgery only	41 (11.2)	159 (17.2)
Surgery followed by adjuvant treatment	161 (44.0)	547 (59.2)
Neoadjuvant treatment followed by surgery and post-neoadjuvant treatment	160 (43.7)	183 (19.8)
Type of surgery, *n* (%)		
Unilateral lumpectomy	127 (34.7)	498 (53.9)
Unilateral mastectomy	128 (35.0)	166 (18.0)

* High-risk eBC was defined using pathologic staging as follows: patients with nodal involvement (T0–4, N1–3, M0); or tumor size > 5 cm (T3–4, N0, M0); or nodal involvement; or by clinical stage IIb, IIc, IIIa, IIIb, or IIIc in patients who received neoadjuvant therapy.

**Table 3:** Clinical characteristics of patients by receipt of neoadjuvant therapy.

	High-Risk * *n* = 366		Non-High-Risk *n* = 924	
	Received Neoadjuvant Therapy	Did Not Receive Neoadjuvant Therapy	Received Neoadjuvant Therapy	Did Not Receive Neoadjuvant Therapy
Overall, *n*	163	203	184	740
ER status, *n* (%)				
Positive	103 (63.2)	151 (74.4)	124 (67.4)	527 (71.2)
Negative	60 (36.8)	51 (25.1)	60 (32.6)	213 (28.8)
Missing	-	1 (0.5)	-	-
PR status, *n* (%)				
Positive	77 (47.2)	117 (57.6)	104 (56.5)	379 (51.2)
Negative	85 (52.1)	85 (41.9)	80 (43.5)	356 (48.1)
Missing, equivocal, or unknown	1 (0.6)	1 (0.5)	-	5 (0.7)
HR status, *n* (%) ^†^				
Positive	109 (66.9)	156 (76.8)	123 (71.7)	534 (73.4)
Negative	54 (33.1)	46 (23.2)	52 (28.3)	197 (26.6)
Missing, equivocal, or unknown	-	1 (0.5)	-	-

* High-risk eBC was defined using pathologic staging as follows: patients with nodal involvement (T0–4, N1–3, M0); or tumor size > 5 cm (T3–4); or clinical stage IIb, IIc, IIIa, IIIb, or IIIc in patients who received neoadjuvant therapy; ^†^ HR status is positive if either the ER or PR status is positive. ER, estrogen receptor; HR, hormone receptor; pCR, pathologic complete response; PR, progesterone receptor.

**Table 4:** Clinical characteristics of patients by receipt of (post-neo)adjuvant therapy.

	High-Risk * n = 366		Non-High-Risk *n* = 924	
	Received (Post-Neo)Adjuvant Therapy	Did Not Receive (Post-Neo)Adjuvant Therapy	Received (Post-Neo)Adjuvant Therapy	Did Not Receive (Post-Neo)Adjuvant Therapy
Overall, *n*	321	45	730	194
ER status, *n* (%)				
Positive	233 (72.6)	21 (46.7)	551 (75.5)	100 (51.5)
Negative	87 (27.1)	24 (53.3)	179 (24.5)	94 (48.5)
Missing	1 (0.3)	-	-	-
PR status, *n* (%)				
Positive	178 (55.5)	16 (35.6)	415 (56.8)	68 (35.1)
Negative	141 (43.9)	29 (64.4)	313 (42.9)	123 (63.4)
Missing, equivocal, or unknown	2 (0.6)	-	2 (0.3)	3 (1.5)
HR status, *n* (%) ^†^				
Positive	241 (75.1)	24 (53.3)	569 (78.0)	106 (54.6)
Negative	79 (24.6)	21 (46.7)	161 (22.0)	88 (45.4)
Missing, equivocal, or unknown	1 (0.3)	-	-	-
Pathologic group stage, *n* (%)				
I	37 (11.5)	3 (6.7)	418 (57.3)	111 (57.2)
II	129 (40.2)	22 (48.9)	124 (17.0)	37 (19.1)
III	43 (13.4)	14 (31.1)	2 (0.3)	5 (2.6)
pCR	69 (21.5)	-	124 (17.0)	25 (12.9)
Unknown	43 (13.4)	6 (13.3)	62 (8.5)	16 (8.2)

* High-risk eBC was defined using pathologic staging as follows: patients with nodal involvement (T0–4, N1–3, M0); or tumor size > 5 cm (T3–4, N0, M0); or nodal involvement; or by clinical stage IIb, IIc, IIIa, IIIb, or IIIc in patients who received neoadjuvant therapy; ^†^ HR status is positive if either the ER or PR status is positive. ER, estrogen receptor; HR, hormone receptor; pCR, pathologic complete response; PR, progesterone receptor.

**Table 5:** Treatment patterns by time period in high-risk and non-high-risk HER2+ eBC.

	High-Risk * *n* = 366									Non-High-Risk ^†^ *n* = 924								
	2011–2013			2014–2017			2018–2021			2011–2013			2014–2017			2018–2021		
	HR+ *n* = 80	HR− *n* = 29	Total N = 109	HR+ *n* = 113	HR− *n* = 33	Total N = 146	HR+ *n* = 72	HR− *n* = 38	Total N = 110	HR+ *n* = 182	HR− *n* = 59	Total ^‡^ N = 241	HR+ *n* = 271	HR− *n* = 98	Total N = 369	HR+ *n* = 222	HR− *n* = 92	Total N = 314
Neoadjuvant therapy, *n* (%)	14 (17.5)	6 (20.7)	20 (18.3)	48 (42.5)	21 (63.6)	69 (47.3)	47 (65.3)	27 (71.1)	74 (67.3)	9 (4.9)	1 (1.7)	10 (4.1)	44 (16.2)	22 (22.4)	66 (17.9)	79 (35.6)	29 (31.5)	108 (34.4)
CT	14 (17.5)	6 (20.7)	20 (18.3)	47 (41.6)	21 (63.6)	68 (46.6)	45 (62.5)	27 (71.1)	72 (65.5)	8 (4.4)	1 (1.7)	9 (3.7)	42 (15.5)	22 (22.4)	64 (17.3)	77 (34.7)	29 (31.5)	106 (33.8)
Platinum-based CT	6 (7.5)	3 (10.3)	9 (8.3)	41 (36.3)	18 (54.5)	59 (40.4)	39 (54.2)	23 (60.5)	62 (56.4)	4 (2.2)	0	4 (1.7)	35 (12.9)	13 (13.3)	48 (13.0)	62 (27.9)	23 (25.0)	85 (27.1)
Taxane	14 (17.5)	6 (20.7)	20 (18.3)	47 (41.6)	21 (63.6)	68 (46.6)	45 (62.5)	27 (71.1)	72 (65.5)	7 (3.8)	1 (1.7)	8 (3.3)	41 (15.1)	21 (21.4)	62 (16.8)	77 (34.7)	29 (31.5)	106 (33.8)
Other CT	6 (7.5)	4 (13.8)	10 (9.2)	3 (2.7)	1 (3.0)	4 (2.7)	3 (4.2)	4 (10.5)	7 (6.4)	4 (2.2)	0	4 (1.7)	2 (0.7)	4 (4.1)	6 (1.6)	3 (1.4)	4 (4.3)	7 (2.2)
ET	0	0	0	3 (2.7)	1 (3.0)	4 (2.7)	8 (11.1)	3 (7.9)	11 (10.0)	1 (0.5)	0	1 (0.4)	2 (0.7)	0	2 (0.5)	5 (2.3)	2 (2.2)	7 (2.2)
HER2 directed ^§^	12 (15.0)	4 (13.8)	16 (14.7)	48 (42.5)	21 (63.6)	69 (47.3)	45 (62.5)	26 (68.4)	71 (64.5)	7 (3.8)	1 (1.7)	8 (3.3)	41 (15.1)	21 (21.4)	62 (16.8)	77 (34.7)	29 (31.5)	106 (33.8)
(Post-neo)adjuvant therapy, *n* (%)	73 (91.3)	16 (55.2)	89 (81.7)	101 (89.4)	29 (87.9)	130 (89.0)	67 (93.1)	34 (89.5)	101 (91.8)	157 (86.3)	35 (59.3)	192 (79.7)	227 (83.8)	62 (63.3)	289 (78.3)	185 (83.3)	64 (69.6)	249 (79.3)
CT	29 (36.3)	7 (24.1)	36 (33.0)	40 (35.4)	9 (27.3)	49 (33.6)	19 (26.4)	6 (15.8)	25 (22.7)	60 (33.0)	30 (50.8)	90 (37.3)	104 (38.4)	36 (36.7)	140 (37.9)	78 (35.1)	30 (32.6)	108 (34.4)
Platinum-based CT	17 (21.3)	4 (13.8)	21 (19.3)	23 (20.4)	2 (6.1)	25 (17.1)	13 (18.1)	3(7.9)	16 (14.5)	38 (20.9)	21 (35.6)	59 (24.5)	49 (18.1)	17 (17.3)	66 (17.9)	16 (7.2)	11 (12.0)	27 (8.6)
Taxane	29 (36.3)	6 (20.7)	35 (32.1)	38 (33.6)	5 (15.2)	43 (29.5)	15 (20.8)	6(15.8)	21 (19.1)	56 (30.8)	29 (49.2)	85 (35.3)	102 (37.6)	36 (36.7)	138 (37.4)	74 (33.3)	29 (31.5)	103 (32.8)
Other CT	9 (11.3)	3 (10.3)	12 (11.0)	15 (13.3)	7 (21.2)	22 (15.1)	4 (5.6)	2(5.3)	6 (5.5)	16 (8.8)	7 (11.9)	23 (9.5)	15 (5.5)	9 (9.2)	24 (6.5)	8 (3.6)	1 (1.1)	9 (2.9)
ET	55 (68.8)	2 (6.9)	57 (52.3)	76 (67.3)	4 (12.1)	80 (54.8)	45 (62.5)	1 (2.6)	46 (41.8)	120 (65.9)	2 (3.4)	122 (50.6)	172 (63.5)	4 (4.1)	176 (47.7)	137 (61.7)	1 (1.1)	138 (43.9)
HER2 directed ^‡^	45 (56.3)	15 (51.7)	60 (55.0)	85 (75.2)	28 (84.8)	113 (77.4)	61 (84.7)	34 (89.5)	95 (86.4)	77 (42.3)	33 (55.9)	110 (45.6)	157 (57.9)	54 (55.1)	211 (57.2)	153 (68.9)	63 (68.5)	216 (68.8)

* 160 (43.7%) patients with high-risk HER2+ eBC received both neoadjuvant and adjuvant therapies; ^†^ 183 (19.8%) patients with non-high-risk HER2+ eBC received both neoadjuvant and adjuvant therapies; ^‡^ One patient in 2011–2013 had an unknown HR status and is not included in the table; ^§^ Other treatments comprising CD4/6 inhibitors, immunotherapies, non-HER2 ADC, anti-angiogenesis drugs, PARP inhibitors, PI3K, TKI, and additional targeted therapies are not shown and may include some HER2-directed therapies. ADC, antibody–drug conjugate; CT, chemotherapy; eBC, early breast cancer; ET, endocrine therapy; HER2, human epidermal growth factor receptor 2; HER2+, HER2-positive; HR, hormone receptor; HR+, hormone receptor-positive; HR−, hormone receptor-negative; PARP, poly-ADP ribose polymerase; PI3K, phosphoinositide 3-kinase; TKI, tyrosine kinase inhibitor.

**Table 6:** Time between/duration of treatment in patients stratified by risk status.

	High-Risk * *n* = 366	Non-High-Risk *n* = 924
Time between/duration of treatment, days (IQR)		
Median time from diagnosis to neoadjuvant therapy	29 (23–43)	34 (27–44)
Median duration of neoadjuvant therapy	145 (132–163)	141 (126–155)
Median time between diagnosis and surgery	76 (26–180)	36 (15–151)
Median time between surgery and (post-neo)adjuvant therapy	31 (17–71)	44 (24–84)

* High-risk eBC was defined using pathologic staging as follows: patients with nodal involvement (T0–4, N1–3, M0); or tumor size > 5 cm (T3–4, N0, M0); or nodal involvement; or by clinical stage IIb, IIc, IIIa, IIIb, or IIIc in patients who received neoadjuvant therapy. IQR, interquartile range.

## Data Availability

The data that support the findings of this study originated from and are the property of Flatiron Health, Inc., which has restrictions prohibiting the authors from making the dataset publicly available. Requests for data sharing by license or by permission for the specific purpose of replicating results in this manuscript can be submitted to PublicationsDataAccess@flatiron.com. The data were deidentified and subject to obligations to prevent reidentification and protect patient confidentiality.

## Appendix A

**Table A1:** Demographic characteristics in HER2+ eBC patients in the sensitivity analysis, overall and stratified by high-risk status.

	Overall (N = 1290)	High-Risk * (*n* = 457)	Non-High-Risk (*n* = 833)
Age at diagnosis			
Mean (SD)	57.6 (12.8)	55.3 (13.3)	58.9 (12.3)
Median [min, max]	58.0 [23.0, 85.0]	55.0 [23.0, 84.0]	59.0 [23.0, 85.0]
Sex, *n* (%) ^†^			
Female	1281 (99.3)	≥ 450 (≥ 98)	827 (99.3)
Male	9 (0.7)	≤ 5 (≤ 1)	6 (0.7)
Race, *n* (%)			
White	817 (63.3)	282 (61.7)	535 (64.2)
Black	133 (10.3)	44 (9.6)	89 (10.7)
Asian	51 (4.0)	15 (3.3)	36 (4.3)
Other	178 (13.8)	76 (16.6)	102 (12.2)
Missing	111 (8.6)	40 (8.8)	71 (8.5)
Ethnicity, *n* (%)			
Hispanic/Latino	104 (8.1)	45 (9.8)	59 (7.1)
Non-Hispanic/Non-Latino	982 (76.1)	344 (75.3)	638 (76.6)
Missing	204 (15.8)	68 (14.9)	136 (16.3)
BMI at diagnosis, *n* (%) ^‡^			
Normal (18.5–< 25)	49 (3.8)	16 (3.5)	33 (4.0)
Overweight (25–< 30)	55 (4.3)	17 (3.7)	38 (4.6)
Obese (≥30)	66 (5.1)	28 (6.1)	38 (4.6)
Missing	1119 (86.7)	396 (86.7)	723 (86.8)
Underweight (< 18.5)	1 (0.1)	0 (0.0)	1 (0.1)
Region, *n* (%)			
South	500 (38.8)	180 (39.4)	320 (38.4)
Northeast	170 (13.2)	56 (12.3)	114 (13.7)
West	171 (13.3)	61 (13.3)	110 (13.2)
Midwest	165 (12.8)	55 (12.0)	110 (13.2)
Missing	284 (22.0)	105 (23.0)	179 (21.5)
Menopausal status, *n* (%)			
Premenopausal	340 (26.4)	153 (33.5)	187 (22.4)
Postmenopausal	829 (64.3)	263 (57.5)	566 (67.9)
Perimenopausal	33 (2.6)	14 (3.1)	19 (2.3)
Missing/not applicable/male	88 (6.8)	27 (5.9)	61 (7.3)

* High-risk eBC was defined using pathologic staging as follows: patients with nodal involvement (T0–4, N1–3, M0); or tumor size > 5 cm (T3–4, N0, M0); or nodal involvement; or by clinical stage IIa, IIb, IIc, IIIa, IIIb, or IIIc in patients who received neoadjuvant therapy; ^†^ To prevent patient identification, values ≤ 5 are masked; ^‡^ BMI calculated as kg/m^2^. BMI, body mass index; SD, standard deviation.

**Table A2:** Treatment pathways from eBC diagnosis over the whole study period (2011–2021) in the sensitivity analysis, stratified by risk status.

	High-Risk * *n* = 457	Non-High-Risk *n* = 833
Treatment pathway after diagnosis, *n* (%)		
No treatment	1 (0.2)	31 (3.7)
Neoadjuvant treatment only	0	0
Neoadjuvant treatment followed by surgery only	3 (0.7)	1 (0.1)
Surgery only	41 (9.0)	162 (19.4)
Surgery followed by adjuvant treatment	161 (35.2)	547 (65.7)
Neoadjuvant treatment followed by surgery and post-neoadjuvant treatment	251 (54.9)	92 (11.0)
Type of surgery, *n* (%)		
Unilateral lumpectomy	170 (37.2)	455 (54.6)
Unilateral mastectomy	147 (32.2)	147 (17.6)

* High-risk eBC was defined using pathologic staging as follows: patients with nodal involvement (T0–4, N1–3, M0); or tumor size > 5 cm (T3–4, N0, M0); or nodal involvement; or by clinical stage IIa, IIb, IIc, IIIa, IIIb, or IIIc in patients who received neoadjuvant therapy.

**Table A3:** Neoadjuvant treatments received stratified by risk status and treatment period.

	High-Risk *			Non-High-Risk ^†^		
	2011–2013 (*n* = 20)	2014–2017 (*n* = 69)	2018–2021 (*n* = 74)	2011–2013 (*n* = 10)	2014–2017 (*n* = 66)	2018–2021 (*n* = 108)
Patients receiving neoadjuvant treatment, *n* (%) ^‡^						
Anastrozole	0	1 (1.4)	1 (1.4)	1 (10.0)	2 (3.0)	3 (2.8)
Carboplatin	9 (45.0)	59 (85.5)	62 (83.8)	4 (40.0)	48 (72.7)	85 (78.7)
Cisplatin	1 (5.0)	0	0	0	0	0
Cyclophosphamide	9 (45.0)	4 (5.8)	7 (9.5)	4 (40.0)	4 (6.1)	7 (6.5)
Docetaxel	10 (50.0)	65 (94.2)	63 (85.1)	6 (60.0)	56 (84.8)	87 (80.6)
Doxorubicin	9 (45.0)	3 (4.3)	6 (8.1)	4 (40.0)	3 (4.5)	7 (6.5)
Etoposide	1 (5.0)	0	0	0	0	0
Exemestane	0	0	1 (1.4)	0	0	0
Fulvestrant	0	1 (1.4)	1 (1.4)	0	0	0
Goserelin	0	0	6 (8.1)	0	0	1 (0.9)
Letrozole	0	1 (1.4)	1 (1.4)	0	0	0
Leuprolide	0	1 (1.4)	2 (2.7)	0	0	3 (2.8)
Mesna	0	0	1 (1.4)	0	0	0
Paclitaxel	10 (50.0)	6 (8.7)	11 (14.9)	3 (30.0)	7 (10.6)	22 (20.4)
Paclitaxel protein-bound	1 (5.0)	2 (2.9)	0	0	1 (1.5)	1 (0.9)
Pertuzumab	2 (10.0)	67 (97.1)	65 (87.8)	3 (30.0)	57 (86.4)	87 (80.6)
Pertuzumab, trastuzumab and hyaluronidase-zzxf	0	0	5 (6.8)	0	0	2 (1.9)
Rituximab	0	0	1 (1.4)	0	0	0
Tamoxifen	0	0	0	0	2 (3.0)	1 (0.9)
Trastuzumab	16 (80.0)	69 (100.0)	44 (59.5)	8 (80.0)	62 (93.9)	55 (50.9)
Trastuzumab-anns	0	0	22 (29.7)	0	0	44 (40.7)
Trastuzumab-dkst	0	0	1 (1.4)	0	0	5 (4.6)
Trastuzumab-dttb	0	0	0	0	0	1 (0.9)
Trastuzumab-qyyp	0	0	5 (6.8)	0	0	8 (7.4)
Trastuzumab and hyaluronidase-oysk	0	0	2 (2.7)	0	0	0
Tretinoin	0	0	0	0	1 (1.5)	0
Vincristine	0	0	0	0	1 (1.5)	0

* 160 (43.7%) patients with high-risk HER2+ eBC received both neoadjuvant and adjuvant therapies; ^†^ 183 (19.8%) patients with non-high-risk HER2+ eBC received both neoadjuvant and adjuvant therapies; ^‡^ Treatments were not mutually exclusive meaning that patients could receive multiple neoadjuvant treatments; therefore, the percentage for each treatment period may total > 100%; percentages in this table are calculated among patients who received neoadjuvant therapy, rather than by overall patient numbers; treatments included biosimilar drugs (e.g., trastuzumab-anns is biosimilar to trastuzumab).

**Table A4:** (Post-neo)adjuvant treatments received stratified by risk status and treatment period.

	High-Risk *			Non-High-Risk ^†^		
	2011–2013 (*n* = 90)	2014–2017 (*n* = 130)	2018–2021 (*n* = 101)	2011–2013 (*n* = 192)	2014–2017 (*n* = 289)	2018–2021 (*n* = 249)
Patients receiving specific (post-neo)adjuvant treatment, *n* (%) ^‡^						
Ado-trastuzumab emtansine	0	0	21 (20.8)	0	0	29 (11.6)
Anastrozole	37 (41.1)	49 (37.7)	31 (30.7)	89 (46.4)	122 (42.2)	93 (37.3)
Bendamustine	0	0	1 (1.0)	0	0	0
Bevacizumab	0	0	0	0	1 (0.3)	0
Bevacizumab-awwb	0	0	0	1 (0.5)	0	0
Blinatumomab	0	0	0	0	1 (0.3)	0
Bortezomib	0	0	0	0	1 (0.3)	0
Capecitabine	0	0	1 (1.0)	1 (0.5)	0	0
Carboplatin	19 (21.1)	25 (19.2)	16 (15.8)	59 (30.7)	66 (22.8)	26 (10.4)
Cisplatin	2 (2.2)	1 (0.8)	0	0	0	0
Cyclophosphamide	10 (11.1)	20 (15.4)	6 (5.9)	22 (11.5)	21 (7.3)	7 (2.8)
Daratumumab and hyaluronidase-fihj	0	0	0	0	1 (0.3)	0
Docetaxel	25 (27.8)	26 (20.0)	18 (17.8)	67 (34.9)	72 (24.9)	29 (11.6)
Doxorubicin	8 (8.9)	14 (10.8)	5 (5.0)	13 (6.8)	15 (5.2)	4 (1.6)
Doxorubicin pegylated liposomal	0	0	0	1 (0.5)	0	0
Durvalumab	0	1 (0.8)	0	0	0	0
Epirubicin	1 (1.1)	4 (3.1)	0	0	1 (0.3)	1 (0.4)
Etoposide	1 (1.1)	0	0	0	0	0
Exemestane	13 (14.4)	17 (13.1)	6 (5.9)	20 (10.4)	32 (11.1)	28 (11.2)
Fluorouracil	0	5 (3.8)	0	5 (2.6)	2 (0.7)	3 (1.2)
Fulvestrant	0	0	1 (1.0)	0	1 (0.3)	0
Gemcitabine	1 (1.1)	1 (0.8)	0	1 (0.5)	1 (0.3)	1 (0.4)
Goserelin	1 (1.1)	8 (6.2)	9 (8.9)	0	3 (1.0)	5 (2.0)
Hydroxyurea	0	0	0	0	0	1 (0.4)
Inotuzumab ozogamicin	0	0	0	0	1 (0.3)	0
Irinotecan	0	0	0	0	1 (0.3)	1 (0.4)
Lapatinib	0	1 (0.8)	0	0	0	0
Lenalidomide	0	0	1 (1.0)	0	1 (0.3)	0
Lenvatinib	0	0	0	0	1 (0.3)	0
Letrozole	22 (24.4)	31 (23.8)	15 (14.9)	41 (21.4)	73 (25.3)	51 (20.5)
Leucovorin	0	0	0	0	0	1 (0.4)
Leuprolide	3 (3.3)	5 (3.8)	5 (5.0)	0	2 (0.7)	5 (2.0)
Levoleucovorin	0	0	0	0	1 (0.3)	0
Mercaptopurine	0	0	0	0	1 (0.3)	0
Methotrexate	0	1 (0.8)	1 (1.0)	1 (0.5)	2 (0.7)	1 (0.4)
Neratinib	0	5 (3.8)	12 (11.9)	0	6 (2.1)	6 (2.4)
Niraparib	0	0	0	1 (0.5)	0	0
Nivolumab	0	1 (0.8)	0	0	0	0
Olaparib	0	0	0	0	0	1 (0.4)
Oxaliplatin	0	0	0	0	1 (0.3)	1 (0.4)
Paclitaxel	11 (12.2)	20 (15.4)	3 (3.0)	20 (10.4)	70 (24.2)	76 (30.5)
Paclitaxel protein-bound	1 (1.1)	1 (0.8)	0	0	3 (1.0)	4 (1.6)
Pegaspargase	0	0	0	0	1 (0.3)	0
Pembrolizumab	0	0	0	0	0	1 (0.4)
Pemetrexed	0	0	0	0	0	1 (0.4)
Pertuzumab	1 (1.1)	26 (20.0)	50 (49.5)	0	22 (7.6)	60 (24.1)
Pertuzumab, trastuzumab and hyaluronidase-zzxf	0	0	5 (5.0)	0	0	2 (0.8)
Polatuzumab vedotin-piiq	0	0	1 (1.0)	0	0	0
Ramucirumab	0	0	0	0	1 (0.3)	0
Rituximab	0	0	1 (1.0)	0	0	0
Rituximab and hyaluronidase	0	0	1 (1.0)	0	0	0
Ruxolitinib	0	0	0	0	0	1 (0.4)
Tamoxifen	37 (41.1)	42 (32.3)	18 (17.8)	46 (24.0)	88 (30.4)	57 (22.9)
Topotecan	0	0	0	1 (0.5)	0	0
Toremifene	1 (1.1)	1 (0.8)	0	2 (1.0)	0	0
Trastuzumab	60 (66.7)	113 (86.9)	54 (53.5)	110 (57.3)	211 (73.0)	120 (48.2)
Trastuzumab-anns	0	1 (0.8)	33 (32.7)	0	0	87 (34.9)
Trastuzumab-dkst	0	0	2 (2.0)	0	0	7 (2.8)
Trastuzumab-qyyp	0	1 (0.8)	8 (7.9)	0	0	25 (10.0)
Tretinoin	0	0	0	1 (0.5)	0	0
Triptorelin	0	1 (0.8)	0	0	0	0
Vincristine	0	0	1 (1.0)	0	1 (0.3)	0
Vinorelbine	0	1 (0.8)	0	0	0	0

* 160 (43.7%) patients with high-risk HER2+ eBC received both neoadjuvant and adjuvant therapies; ^†^ 183 (19.8%) patients with non-high-risk HER2+ eBC received both neoadjuvant and adjuvant therapies; ^‡^ Treatments were not mutually exclusive meaning that patients could receive multiple neoadjuvant treatments; therefore, the percentage for each treatment period may total > 100%; percentages in this table are calculated among patients who received (post-neo)adjuvant therapy, rather than by overall patient numbers; treatments included biosimilar drugs (e.g., trastuzumab-anns is biosimilar to trastuzumab).
